# DNA Hypermethylation of the *ZNF382* Promoter Region and Low mRNA Expression of *ZNF382* Promote Diffuse Large B‐Cell Lymphoma Occurrence and Progression

**DOI:** 10.1002/cnr2.70502

**Published:** 2026-02-19

**Authors:** Wanhua An, Shuli Guo, Sizhe Liu, Pengli Xiao, Emma Mi, Huirui Wang

**Affiliations:** ^1^ Department of Hematology Luoyang Central Hospital Affiliated to Zhengzhou University Luoyang China; ^2^ Lincoln University College Petaling Jaya Malaysia; ^3^ Nuffield Department of Primary Care Health Sciences Radcliffe Observatory Quarter, University of Oxford Oxford UK

**Keywords:** decitabine, diffuse large B‐cell lymphoma, methylation, ZNF382

## Abstract

**Background:**

The pathogenesis of diffuse large B‐cell lymphoma (DLBCL) remains unclear. Zinc finger protein 382 (*ZNF382*) expression is significantly downregulated in DLBCL, which is associated with poor prognosis. However, the underlying mechanisms remain unknown.

**Aims:**

To investigate the association between DNA methylation of the promoter region of *ZNF382* and *ZNF382 expression* with the occurrence and progression of DLBCL and to analyze the clinical significance of *ZNF382* in DLBCL.

**Methods and Results:**

DLBCL cell lines and reactive hyperplastic lymph node tissues were used as the experimental subjects. Methylation‐specific polymerase chain reaction and reverse‐transcription polymerase chain reaction analyses revealed significantly higher methylation of the promoter region of *ZNF382* (*p* < 0.0001), whereas *ZNF382* mRNA expression was significantly lower (*p* < 0.01) in DLBCL cells compared with those in reactive hyperplastic lymph node tissues. Cells were treated with the demethylating agent decitabine (DAC), and the methylation status of the promoter and expression levels of *ZNF382* were determined. *ZNF382* promoter methylation was significantly reduced (*p* < 0.01) in DLBCL cells, whereas *ZNF382* mRNA expression was significantly increased (*p* < 0.01) compared with those in the control cells. Furthermore, compared with that in the blank control group, the apoptosis rate of DLBCL cells was significantly increased following DAC intervention (*p* < 0.01). A cell model of *ZNF382* overexpression was constructed, and its proliferation, migration, and clonogenic capacities were detected using CCK‐8, Transwell, and soft agar assays, respectively. Compared with those of the vector control group, the cell proliferation, migration, and clone formation abilities of the *ZNF382* overexpression group were significantly inhibited (*p* < 0.01).

**Conclusion:**

DNA hypermethylation in the promoter region of *ZNF382* and low *ZNF382* mRNA expression are closely related to the occurrence and progression of DLBCL. Moreover, *ZNF382* overexpression significantly increased apoptosis and inhibited cell proliferation, migration, and clone formation. Therefore, *ZNF382* has potential as a therapeutic target in the treatment of DLBCL.

## 1 Introduction

Diffuse large B‐cell lymphoma (DLBCL), a type of non‐Hodgkin lymphoma (NHL), is highly malignant and has a high incidence rate [1]. It accounts for about one‐third of all NHL cases, and its incidence is increasing. Despite the efficacy of the R‐CHOP first‐line chemotherapy regimen, up to 50% of patients with relapsed or refractory DLBCL have an extremely poor prognosis, with a 2‐year overall survival (OS) of 20%–40% [2, 3]. Therefore, in‐depth research on the pathogenesis of DLBCL is urgently needed. Such research has valuable clinical significance, as it may provide new strategies for the diagnosis and treatment of lymphomas.

ZNF382 is a zinc finger protein family isoform. Previously, our research group has demonstrated that ZNF382 expression is significantly downregulated in the lymph node tissues of patients with DLBCL and is associated with the prognosis of the disease [4]. However, the correlation between ZNF382 and DLBCL remains unknown.

Therefore, the present study aimed to explore the effects of ZNF382 on cell apoptosis, proliferation, migration, and cloning in DLBCL cell lines. To this end, we investigated the methylation status of the promoter region, the level of mRNA expression of *ZNF38*2, and the impact of interventions with demethylation drugs. This study provides a theoretical basis for understanding the pathogenesis of DLBCL and identifying new diagnostic and therapeutic strategies.

## 2 Materials and Methods

### 2.1 Patient Tissue Collection

All patients provided written informed consent, and the experiments were reviewed and approved by the Medical Ethics Committee of Luoyang Central Hospital (Approval No. LWLL‐2022‐02‐11). Lymph node tissues were obtained from 20 patients diagnosed with DLBCL between January 1, 2014, and December 31, 2018. Lymph node biopsy specimens were collected from patients with a pathologic diagnosis of reactive hyperplasia of lymph nodes for ex vivo experiments.

### 2.2 Cell Culture

OCI‐LY10 and U2932 cells (Shanghai Yu Bo Biotech Co. Ltd., China) were cultured in sterile RPMI‐1640 medium (HyClone, Logan, UT, USA) containing 15% fetal bovine serum (FBS; HyClone) and 1% penicillin‐streptomycin, while 293 T cells were sterile‐cultured in high‐glucose Dulbecco's modified Eagle's medium (DMEM; HyClone) containing 15% FBS and 1% penicillin‐streptomycin. The cells were incubated in a constant temperature incubator at 37°C, 5% CO_2_, and 95% saturated humidity. OCI‐LY10 and U2932 cells were subcultured at a ratio of 1:1.5 to 1:2, respectively, every 1–2 days. The 293T cells were subcultured at a ratio of 1:2 to 1:3 when the cell density reached 75%–85%.

### 2.3 Demethylation Drug Treatment

Cells exhibiting good growth were seeded into 6‐well culture plates at a concentration of 3 × 10^5^/mL, with 2 mL per well, and a blank control and 1, 2, and 4 μmol/L DAC groups were established. Half of the culture medium was changed every 24 h to maintain a stable drug concentration. After 96 h, the cells were collected for RNA and DNA extraction for subsequent experiments.

### 2.4 Methylation‐Specific PCR Amplification (MSP) Analysis

Cell collection, DNA extraction, and bisulfite modification of DNA were performed according to the instructions of the Gene methylation‐specific PCR [MSP] amplification kit (Cloud‐Clone Corp., Wuhan). The reaction conditions were as follows: (i) 94°C for 1 min and 94°C for 30 s, one cycle; (ii) 60°C (M)/58°C (U) for 30 s and 72°C for 30 s, 40 cycles; and (iii) 72°C for 10 min, one cycle. The amplification products were separated via DNA agarose gel electrophoresis, and gel imaging was performed to enable the analysis of the results. The absorbance values of the methylated and unmethylated bands were measured using Image Pro Plus 6.0 to calculate grayscale values. The ratio of the two values (M grayscale value/M grayscale value + U grayscale value) was considered the methylation level. The primer sequences used in this study (designed by Shanghai Sangon Biotech Company, Shanghai, China) are presented in Table 1.

**TABLE 1:** Sequences of the primers used in this study.

Assay	ID	Forward sequence	Reverse sequence	Product length/bp
MSP	ZNF382(M)	5′‐GGCGATTAACGGGTCGTTTC‐3′	5′‐AAAATTTCCAAACCCGACTCG‐3′	230
	ZNF382(U)	5′‐GTGGTGATTAATGGGTTGTTTT‐3′	5′‐CAAAATTTCCAAACCCAACTCA‐3′	233
RT‐PCR	ZNF382	5′‐CCTTACAGGGATCAGTGTCA‐3′	5′‐CAACTTGCGGATCATATCAG‐3′	173
	GAPDH	5′‐CCAGCAAGAGCACAAGAGGAA‐3′	5′‐CAAGGGGTCTACATGGCAACT‐3′	114

Abbreviations: ID, the name of a gene; M, methylated; MSP, methylation‐specific PCR; RT‐PCR, reverse transcription polymerase chain reaction; U, unmethylated.

### 2.5 RT‐PCR Analysis

Total RNA was extracted from cells using Trizol (Invitrogen, Carlsbad, CA, USA) reagent, and cDNA was synthesized from the RNA using reverse transcriptase. Gene sequences were obtained from GenBank, and primers were designed using Primer 5.0 software. Amplification was performed by pre‐heating samples at 95°C for 1 min, followed by denaturation at 95°C for 30 s, annealing at 58°C (*GAPDH*)/50°C (*ZNF382*) for 30 s, and extension at 72°C for 30 s and 72°C for 10 min. Both *GAPDH* and the target gene were amplified, requiring 27 cycles and 36 cycles, respectively. The PCR products were identified using agarose gel electrophoresis with a mass fraction of 2%. The experiment was repeated three times, and *GAPDH* was used as the internal reference to quantify the relative expression of the target gene. Semi‐quantitative analysis was performed using the gel image analysis software Image Pro Plus 6.0. Relative expression indicated the expression level of the target gene, expressed as a fold‐change compared to the level of the reference gene. The primer sequences used are presented in Table 1.

### 2.6 Apoptosis Analysis

The Annexin V‐FITC/PI double staining method and flow cytometry were performed to measure the apoptotic rate of cells. Cells were divided into blank control and 1, 2, and 4 μmol/L DAC groups, and the effect of different DAC concentrations on cellular apoptosis was measured after 72 h. The assays were performed in triplicate (biological replicates). The apoptotic rate was calculated by summing the rate of cell population of the FITC+PI− and the FICT+PI+ groups.

### 2.7 Construction of ZNF382 Stable Expression Cell Lines

The 293T cells were divided into vector (empty vector virus) and stably transfected ZNF382 cell groups. Empty shuttle and packaging plasmids were added to the vector group. Shuttle (pCDH‐CMV‐GFP‐ZNF382, Figure 5a) and packaging (pSPAX2, pMD2.G) plasmids (designed by the Shanghai Sangon Biotech company, Shanghai, China) containing *ZNF382* were added to stably transfect cells with ZNF382, and LVtransm transfection reagent (TaKaRa Bio, Shiga, Japan) was added. Subsequently, 293 T cells were co‐transfected using the lentivirus packaging method, and the lentivirus was removed at 24, 48, and 72 h after transfection. The viral titer was 5 × 10^11^ TU·L^−1^. According to the lentivirus transfection pre‐experiment results, the multiplicity of infection was 100. The DLBCL cell line was transfected based on the viral titers, which were determined using the dilution ratio method. The cell line was seeded into 24‐well plates, and polybrene working solution and concentrated virus were added. Puromycin was used to screen stable transgenic strains. Stable cells were identified using flow cytometry after 72 h (Figure 5e).

### 2.8 Cell‐Counting Kit‐8 (CCK‐8) Assay

Cells were divided into the vector control (empty vector virus) and stable ZNF382 expression groups. Cells were inoculated (1 × 10^4^ cells per well) onto a 96‐well plate. CCK‐8 (Solarbio, Beijing, China) solution was added to each well at 0, 24, 48, and 72 h. Subsequently, the optical density (OD) value at 490 nm was measured, and the cell proliferation inhibition rate at 0.5, 1, 2, and 4 h of cultivation was calculated using the following formula: Cell proliferation inhibition rate = [1 − (A − C)/(B − C)] × 100% (absorbance of test well = A, absorbance of control well = B, absorbance of blank well = C). The assays were performed in triplicate (biological replicates).

### 2.9 Transwell Assay

Vector control (empty vector virus) and stably transfected ZNF382 groups were established. DLBCL cells were selected, centrifuged, resuspended, and counted. Then, the cell concentration was adjusted to 2 × 10^5^ cells/mL, and 600 μL of 1640 culture medium containing 15% FBS was added to a 24‐well plate chamber. Subsequently, 200 μL of cell suspension (i.e., 4 × 10^4^ cells) was added to the small chamber. The cells were cultivated for 72 h, the chamber was removed, and a representative field of view under an inverted microscope was selected for photography and cell counting. The assays were performed in triplicate (biological replicates).

### 2.10 Colony Formation Assay

The vector control (empty vector virus) and stably transfected ZNF382 groups were used for this experiment. Bottom agar medium was added to 6‐well plates at 1.5 mL/well. After solidification, the cells were resuspended in 0.3% upper agar medium and seeded into the same plates, with two secondary wells for each group. After solidification, 1640 medium with 15% FBS was added to the surface of the upper medium. The surface medium was changed daily, and the culture was suspended until colonies visible to the naked eye formed. Polyformaldehyde was added to fix cells, which were then stained with crystal violet dye. Clone balls with a diameter greater than 50 μm were counted, and photographs were taken. The assays were performed in triplicate (biological replicates).

### 2.11 Statistical Analysis

Data are expressed as the mean ± SD and were analyzed using analysis of variance (ANOVA) followed by a post hoc Newman–Keuls test or Student's *t*‐test as appropriate (SPSS 22.0, Chicago, IL, USA; and GraphPad Prism 9.0, GraphPad Inc., La Jolla, CA, USA). A *t*‐test was used for inter‐group comparisons, and one‐way ANOVA was used for multi‐group comparisons. *P*‐values < 0.05 were considered statistically significantly different.

## 3 Results

### 3.1 Promoter Methylation of ZNF382 Was Significantly Increased in DLBCL Cells

**FIGURE 1:**
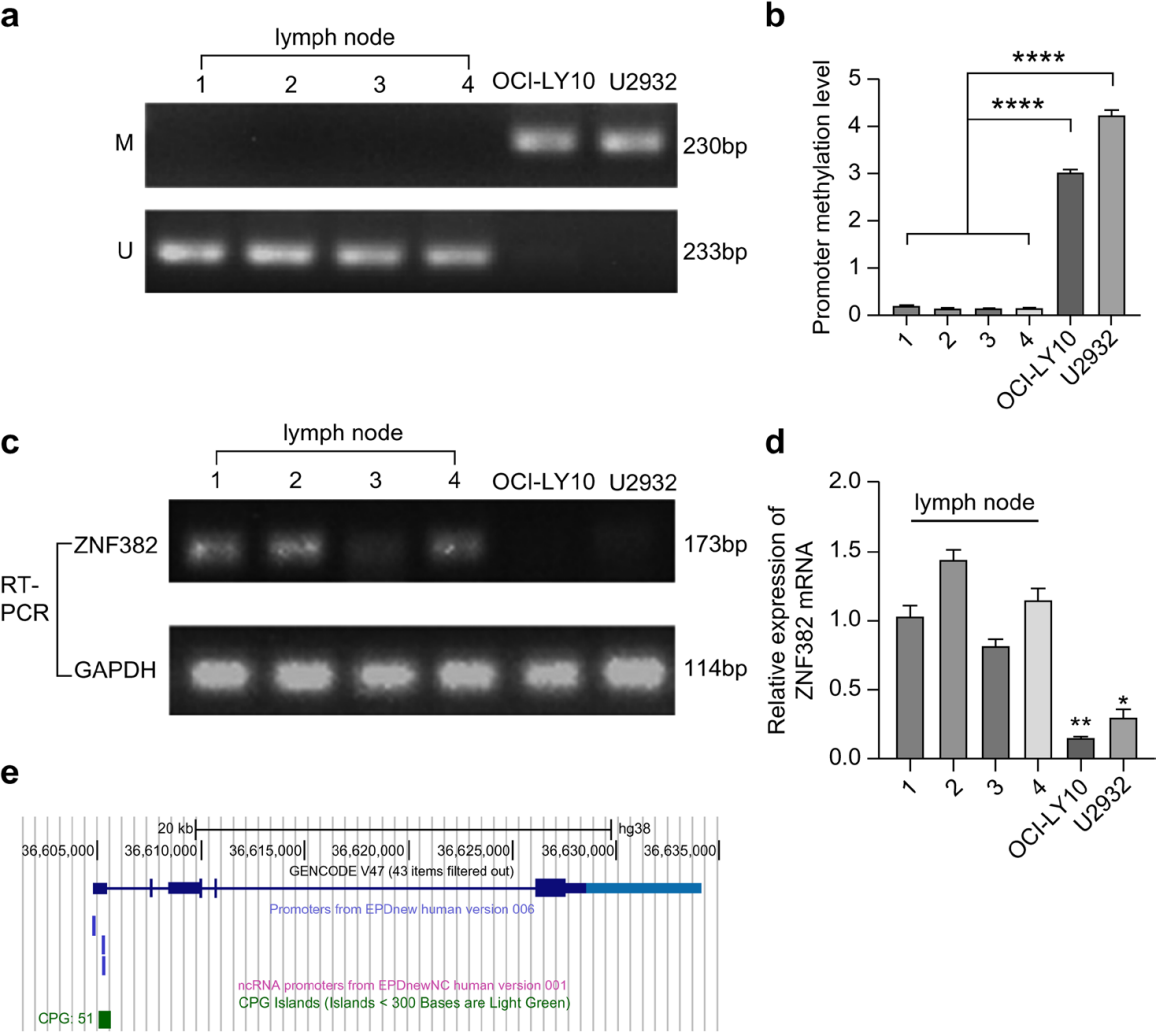
Methylation levels of the *ZNF382* promoter and expression levels of *ZNF382* mRNA. (a, b) Promoter methylation level of *ZNF382* in DLBCL cell lines. (c, d) Expression levels of *ZNF382* mRNA in DLBCL cell lines. Lymph node (1, 2, 3, 4): Lymph node tissue samples of the control group; M: Methylated; U: Unmethylated. Error bars represent the mean ± SD (*n* = 3). *p < 0.05; **p < 0.01; ****p < 0.0001. *GAPDH* was used as the reference gene to calculate the relative expression of *ZNF382*. (e) A schematic representation of the promoter of the *ZNF382* gene (blue vertical bar) and CpG island (red vertical bar). Note: We searched for the gene structure of ZNF382 using the UCSC Genome Browser (https://genome.ucsc.edu/index.html) to obtain the predicted location of its CpG island and promoter. The primer sequences used in this study (designed by Shanghai Sangon Biotech company, Shanghai, China) are presented in Table 1.

Methylation of the *ZNF382* promoter region in reactive hyperplastic lymph node tissues and OCI‐LY10 and U2932 cells was assessed using an MSP assay. The experimental results revealed that methylation of the *ZNF382* promoter region was significantly increased in both DLBCL cell lines compared to that in reactive hyperplastic lymph node tissues (*p* < 0.0001; Figure 1a,b). As *ZNF382* promoter hypermethylation serves as a cell line‐specific epigenetic marker in DLBCL, this difference between clinical cohorts and in vitro models supports the translational potential of *ZNF382* methylation profiling for patient stratification.

### 3.2 ZNF382 mRNA Expression Was Significantly Decreased in DLBCL Cells

RT‐PCR was used to detect the expression levels of *ZNF382* mRNA in reactive proliferative lymph node tissues and OCI‐LY10 and U2932 cells. Based on the results of triplicate experiments, the expression levels of *ZNF382* mRNA were significantly reduced in OCI‐LY10 (*p* < 0.01) and U2932 cells (*p* < 0.01) compared to those in reactive proliferative lymph node tissues (Figure 1c,d). In addition, the expression levels of ZNF382 were assessed in the lymph nodes obtained from patients with DLBCL and healthy donors. The results revealed that ZNF382 expression was significantly higher in the healthy group compared with that in the patients with DLBCL (0.1236 vs. 0.0211, *p* < 0.001, Table S1 and Figure S1). We searched for the gene structure of ZNF382 using the UCSC Genome Browser (https://genome.ucsc.edu/index.html) to obtain the predicted location of its CpG island and promoter (Figure 1e).

### 3.3 Effect of the Hypomethylation Agent DAC on the Methylation Level of the ZNF382 Promoter in DLBCL Cells

**FIGURE 2:**
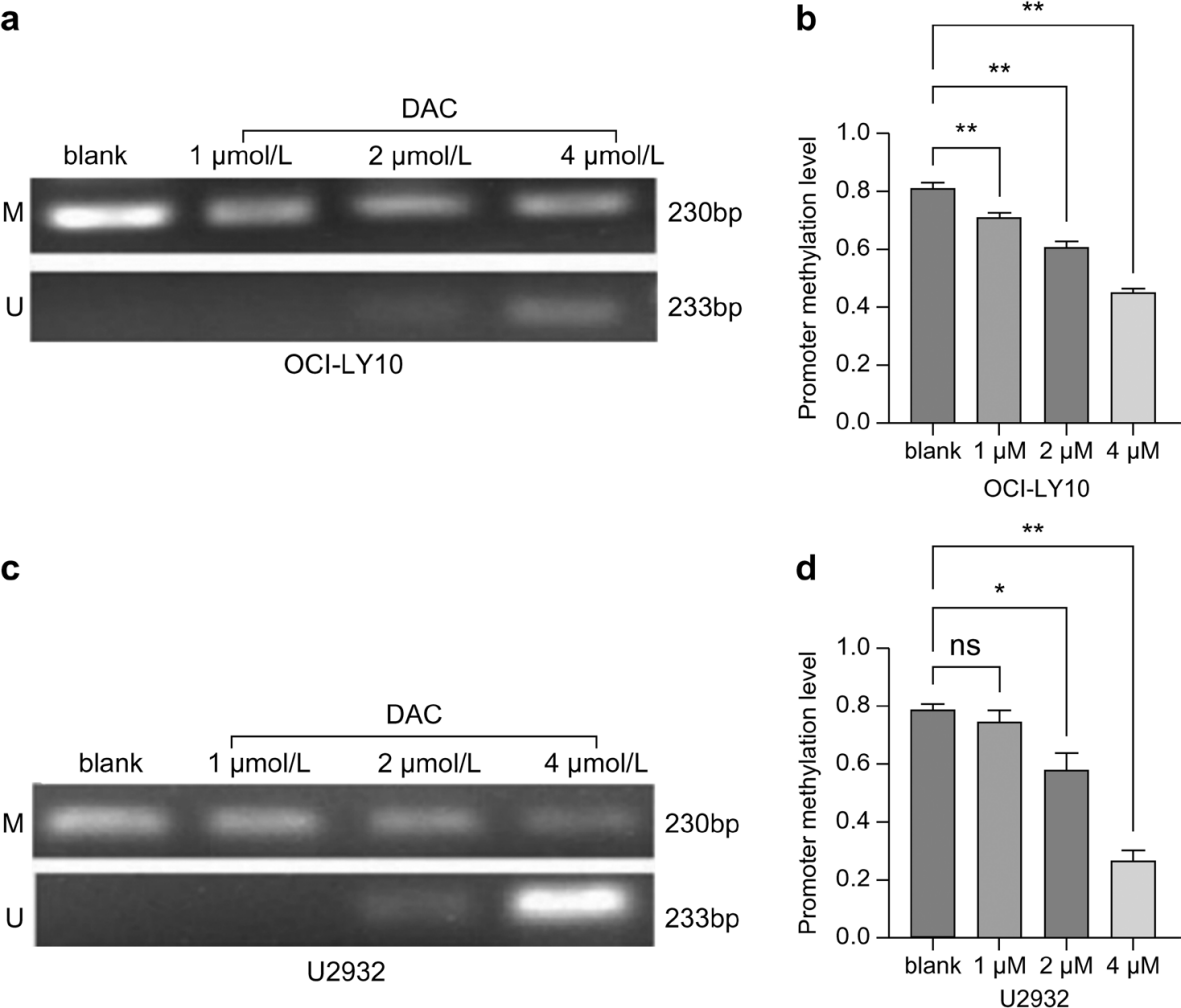
Effects of the hypomethylation agent DAC on the methylation level of the *ZNF382* promoter in (a, b) OCI‐LY10 and (c, d) U2932 cells (**p < 0.01; *p < 0.05). *GAPDH* was used as the reference gene to calculate the relative expression of *ZNF382*. Error bars represent the mean ± SD (*n* = 3).

The MSP assay indicated that *ZNF382* promoter methylation was significantly lower in OCI‐LY10 cells treated with 1, 2, and 4 μmol/L DAC compared with that in the blank control group after 96 h of DAC intervention (*p* < 0.01). However, its methylation levels in U2932 cells were not significantly different between the 1 μmol/L DAC and blank control groups (*p* > 0.05), whereas those in the 2 μmol/L (*p* < 0.05) and 4 μmol/L (*p* < 0.01) DAC groups were markedly decreased (Figure 2a–d). Notably, an abnormally decreased methylation level was observed in cells treated with 4 μmol/L DAC. This suggests that increased DAC concentrations may induce aberrant metabolic activity, potentially leading to mRNA degradation and subsequent underestimation of target expression in assays. This finding indicates the need for caution in future clinical applications of DAC, particularly regarding dosage optimization to balance efficacy and potential off‐target effects.

### 3.4 Effects of the Hypomethylation Agent DAC on ZNF382 mRNA Expression in DLBCL Cells

**FIGURE 3:**
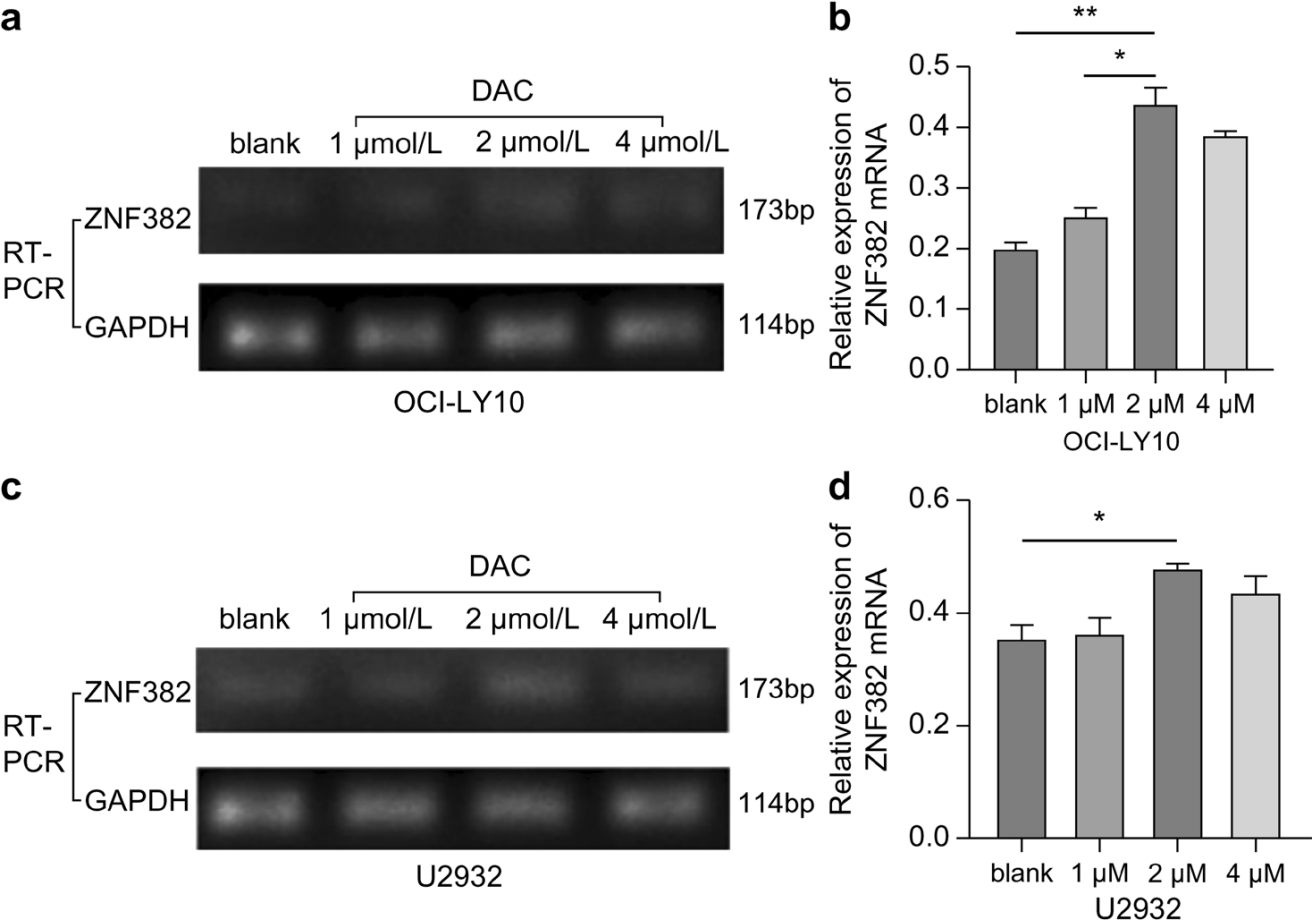
Effects of the hypomethylation agent DAC on *ZNF382* mRNA expression in (a, b) OCI‐LY10 and (c, d) U2932 cells (*p < 0.05; **p < 0.01). *GAPDH* was used as the reference gene to calculate the relative expression of *ZNF382*. Error bars represent the mean ± SD (*n* = 3).

RT‐PCR analysis revealed that in OCI‐LY10 cells treated with 2 μmol/L DAC, *ZNF382* mRNA expression was markedly increased compared with that in the blank control group (*p* < 0.01; Figure 3a,b). Similarly, *ZNF382* mRNA expression in U2932 cells treated with 2 μmol/L DAC was significantly increased (*p* < 0.05; Figure 3c,d). However, there was no significant difference in *ZNF382* mRNA expression in OCI‐LY10 and U2932 cells between the 1 or 4 μmol/L DAC groups and the blank control group (*p* > 0.05; Figure 3a–d).

### 3.5 Hypomethylation Agent DAC Induced Cell Apoptosis

**FIGURE 4:**
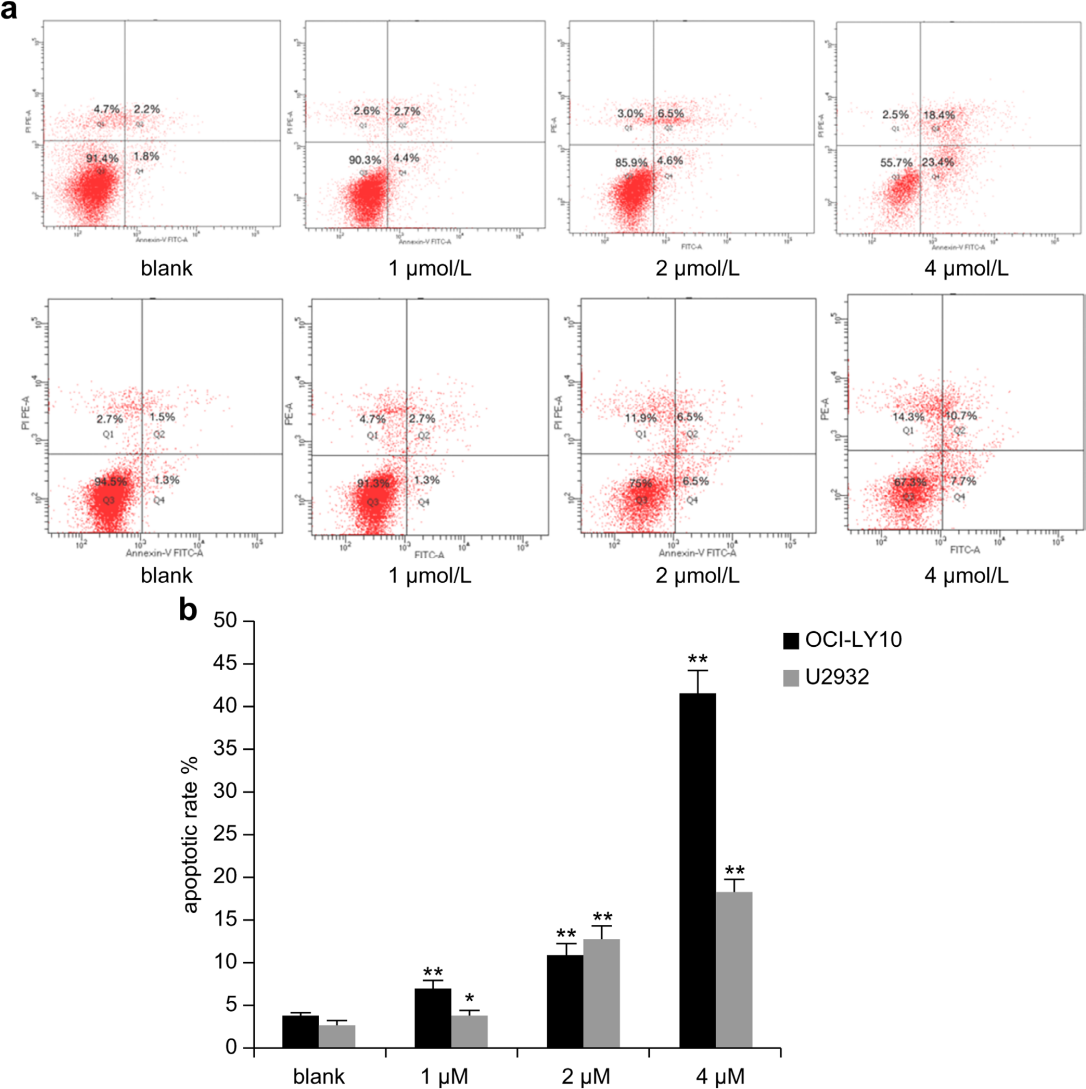
Effects of DAC on DLBCL cell apoptosis. (a) The apoptotic rate in DLBCL cell lines treated with different concentrations of DAC; (b) Comparison of the apoptotic rates in cells treated with different concentrations of DAC and that of the blank control group (*p < 0.05; **p < 0.01). Error bars represent the mean ± SD (*n* = 3).

Cell apoptosis was evaluated using flow cytometry. After treatment with DAC for 72 h, the apoptotic rate in cells increased as drug concentration increased (Figure 4a). In OCI‐LY10 cells, the apoptotic rate of the DAC groups was significantly higher than that of the blank control group (*p* < 0.01). In U2932 cells, the apoptotic rate in the 1 μmol/L DAC group was significantly increased compared with that in the blank control group (*p* < 0.05), with the most pronounced increase observed in the 2 and 4 μmol/L DAC groups (*p* < 0.01; Figure 4b).

### 3.6 Overexpression of ZNF382 Suppressed the Proliferation of DLBCL Cells

**FIGURE 5:**
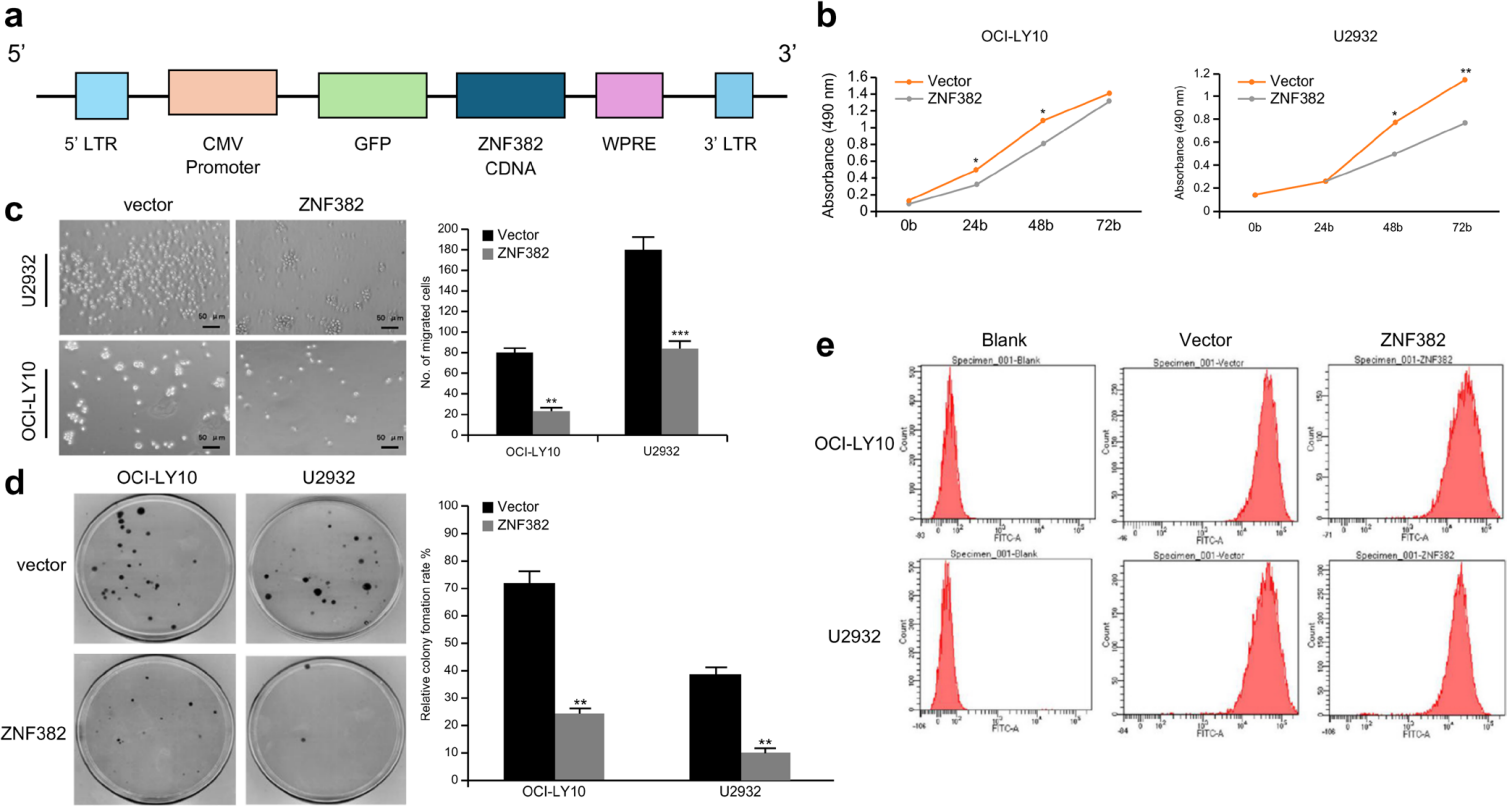
Effect of ZNF382 on the biological function of DLBCL cells. (a) Construction of the *ZNF382* overexpression plasmid. Effects of *ZNF382* overexpression on cell (b) proliferation, (c) migration, and (d) clone formation. (e) Flow cytometry analysis of cells transfected with the stable *ZNF382* overexpression vector after 72 h of transfection. *p < 0.05; **p < 0.01; ***p < 0.001. Error bars represent the mean ± SD (*n* = 3).

The cell purity of the stably transformed cells was determined using flow cytometry to be almost 100%. Subsequently, after cells were cultured for 24, 48, and 72 h, their cell proliferation abilities were detected using the CCK‐8 assay. The results demonstrated that the proliferation ability of OCI‐LY10 cells overexpressing *ZNF382* was markedly lower than that of the vector group at 24 and 48 h (*p* < 0.05). After 48 h and 72 h of culture, the proliferation ability of the U2932 cell group was significantly lower (*p* < 0.05 and *p* < 0.01, respectively) than that of the vector group (Figure 5b).

### 3.7 Overexpression of ZNF382 Suppresses the Migration of DLBCL Cells

Transwell migration assays were used to evaluate the migratory ability of cells. The results revealed that, compared to that in the vector group, the migration of OCI‐LY10 cells overexpressing *ZNF382* was reduced (*p* < 0.01), and the number of cells that migrated in the U2932 group was more significantly reduced (*p* < 0.001) (Figure 5c).

### 3.8 Overexpression of ZNF382 Suppressed the Clonogenic Ability of DLBCL Cells

Colony formation assays indicated that, compared with that in the vector control group, overexpression of *ZNF382* suppressed the clonogenic ability of both OCI‐LY10 and U2932 cells (*p* < 0.01) (Figure 5d). These findings suggest that *ZNF382* acts as a prominent tumor suppressor gene, and its epigenetic silencing (e.g., via promoter hypermethylation) significantly impairs the viability of DLBCL cells and may contribute to lymphomagenesis.

## 4 Discussion

Zinc finger proteins were first discovered in *Xenopus* oocytes. They represent the largest transcription factor family in mammals, with approximately 800 members [5, 6]. ZNF382 is located at position 19q13.12 and plays a crucial role in cell growth [7]. Many studies on biological functions have indicated that ZNF382 exhibits an anti‐tumor effect on various tumor types, such as liver cancer and gastrointestinal malignancies. The mechanism through which ZNF382 increases cell apoptosis may involve the inhibition of cell proliferation, migration, invasion, colony formation, and tumorigenic effects [8, 9, 10]. However, there are no studies correlating ZNF382 expression with the onset or progression of DLBCL. A previous clinical study by our group [4] confirmed that low ZNF382 protein expression in pathological samples was positively correlated with poor prognosis in patients with DLBCL. This suggests that ZNF382 downregulation may be involved in the onset or progression of DLBCL. Thus, we hypothesized that ZNF382 may play an essential role as a tumor suppressor. In the present study, we demonstrated an association between low ZNF382 protein expression and DLBCL.

Epigenetic modifications, especially DNA methylation and mRNA expression, play a crucial role in the pathogenesis of tumors [11]. The possible molecular mechanisms of DNA methylation‐induced inhibition of gene transcription can be divided into three categories: (1) direct inhibition of the binding of transcription factors due to DNA promoter methylation [12]; (2) recruitment of transcription inhibition complexes following DNA methylation, wherein methylation facilitates protein binding to the DNA, inhibiting the binding of transcription factors or removing histone acetylation [13, 14]; and (3) alteration of chromatin conformation caused by DNA methylation [15]. In recent years, research has demonstrated that epigenetic modifications also have an indispensable role in the occurrence and progression of DLBCL [16]. Our study contributes to existing literature by demonstrating that DNA methylation of the promoter region of *ZNF382* is significantly increased in DLBCL cell lines. As previous studies have indicated, *ZNF382* serves as a tumor suppressor in DLBCL, and our findings are consistent with existing literature demonstrating that tumor suppressor genes are often hypermethylated in tumor tissues [17, 18, 19]. In the present study, RT‐PCR analysis revealed that *ZNF382* mRNA expression was significantly downregulated in DLBCL cell lines. Therefore, we speculate that DNA hypermethylation may inhibit gene transcription and expression through some of the abovementioned mechanisms, potentially contributing to the onset and development of DLBCL.

The demethylating drugs DAC and azacytidine (AZA) are widely used in clinical practice. They are the preferred drugs for treating patients with medium‐ to high‐risk myelodysplastic syndromes, as they can significantly prolong OS in these patients [20]. In recent years, hypomethylating agents have also been utilized for relapsed and/or refractory multiple myeloma and lymphoma. For example, a single‐arm, open‐label, single‐center phase II clinical study demonstrated that low‐dose DAC combined with bortezomib and dexamethasone is a tolerable and effective treatment option for patients with first‐relapsed myeloma [21]. Furthermore, a multi‐center phase I clinical study demonstrated that AZA can improve the safety and efficacy of R‐CHOP in newly diagnosed patients with moderate‐ and high‐risk DLBCL [22]. Han et al. [23] reported that in relapsed and/or refractory classic Hodgkin lymphoma, the complete response rate of patients treated with DAC combined with carrelizumab was significantly higher than that of patients treated with carrelizumab alone. However, the mechanisms of action of demethylating agents in the treatment of lymphoma have not been characterized. Our study demonstrated, at the cellular level, that low expression of *ZNF382* in DLBCL was closely related to the hypermethylation of its promoter region, and its expression was significantly upregulated after intervention with DAC. Further, we found that *ZNF382* overexpression significantly increased the apoptotic rate of tumor cells and significantly inhibited their proliferation, migration, and colony formation abilities. In our experiments, we also observed that as the dosage of DAC increased, the apoptotic rate increased, which may lead to partial degradation of mRNA, in turn causing a gradual decline in the correlation between the expression level of *ZNF382* and the degree of demethylation. Chiou and Chang [24] also found that treatment with DAC induced increased cellular autophagy, leading to the degradation of *β‐TrCP* mRNA. Moreover, we speculate that the insignificant expression trend associated with methylation at a concentration of 4 μmol/L (Figure 3) was mainly because DAC promoted apoptosis, which exacerbated the degradation of *ZNF382* mRNA, rather than being due to other toxic effects. This suggests that high concentrations of DAC may further reduce the expression level of *ZNF382* through a dual mechanism of spontaneous induction of mRNA degradation and exacerbation of apoptosis‐induced mRNA degradation, resulting in partially uncorrelated results in our experiments. However, it remains unclear whether *ZNF382* mRNA undergoes degradation during treatment with DAC. Therefore, we suggest 2 μM may be the optimal dosage for DAC.

A limitation of our study is that the findings were not validated in vivo or in primary DLBCL cell lines. Therefore, further studies are required to confirm the optimal DAC dosage for controlling ZNF382 expression levels.

In summary, this study demonstrates that DNA hypermethylation in the promoter region and low mRNA expression of *ZNF382* are strongly correlated with the progression of DLBCL in vitro. In our future studies, we will use bioinformatics to screen the downstream targets of *ZNF382* and conduct in vivo studies using a mouse transplantation tumor model. Subsequently, our center will strive to clarify the crucial signaling pathways involved in the onset and progression of DLBCL, providing a new theoretical basis along with diagnostic and therapeutic ideas for relapsed and refractory DLBCL.

## Author Contributions

**Wanhua An:** data curation, formal analysis, investigation, methodology, validation, writing – original draft, writing – review and editing. **Shuli Guo:** resources. **Sizhe Liu:** investigation, methodology, writing – original draft. **Pengli Xiao:** software, supervision. **Emma Mi:** visualization. **Huirui Wang:** conceptualization, funding acquisition, project administration, writing – review and editing.

## Funding

The work was financially supported by Department of Hematology, Luoyang Central Hospital—Henan Provincial Key Clinical Specialty; Key Discipline of Hematology Medicine in Luoyang City, Luoyang Central Hospital; Key Research and Development Project of Henan Province: 251111313900; Henan Provincial Science & Technology Project: 222102310049 and Henan Provincial Medical Science and Technology Tackling Program Joint Construction Project: LHGJ20240737.

## Ethics Statement

The study was reviewed and approved by the Medical Ethics Committee of Luoyang Central Hospital (Approval No. LWLL‐2022‐02‐11).

## Consent

All patients provided written informed consent.

## Conflicts of Interest

The authors declare no conflicts of interest.

## Supporting information


**Figure S1:** Immunohistochemistry results demonstrated the differences between healthy lymph nodes (20 cases) and those of patients with DLBCL (57 cases). (a), The high‐expression and (b) low‐expression regions of ZNF382 in the lymph nodes of patients. (c) The corresponding high‐expression and (d) low‐expression regions of ZNF382 in healthy individuals. Immunohistochemistry staining was performed according to the protocol of the two‐step IHC kit (E‐IR‐R215, Elabscience) for sample processing and antibody labeling. ZNF382 antibodies were purchased from Abcam (Catalog No. ab25918).
**Table S1:**. Protein expression levels of ZNF382 in the DLBCL and control group M (quartile).

## Data Availability

The data that support the findings of this study are available from the corresponding author upon reasonable request.
