# Supplementary material for: DNA Hypermethylation of the ZNF382 Promoter Region and Low mRNA Expression of ZNF382 Promote Diffuse Large B‐Cell Lymphoma Occurrence and Progression
Source: Cancer Rep (Hoboken). 2026 Feb 19;9(2):e70502. doi: 10.1002/cnr2.70502 (PMC12920067; doi:10.1002/cnr2.70502)
Supplement: Supplementary file 1 — Figure S1: Immunohistochemistry results demonstrated the differences between healthy lymph nodes (20 cases) and those of patients with DLBCL (57 cases). (a), The high‐expression and (b) low‐expression regions of ZNF382 in the lymph nodes of patients. (c) The corresponding high‐expression and (d) low‐expression regions of ZNF382 in healthy individuals. Immunohistochemistry staining was performed according to the protocol of the two‐step IHC kit (E‐IR‐R215, Elabscience) for sample processing and antibody labeling. ZNF382 antibodies were purchased from Abcam (Catalog No. ab25918). Table S1:. Protein expression levels of ZNF382 in the DLBCL and control group M (quartile). [file CNR2-9-e70502-s001.docx]

**Supporting information**


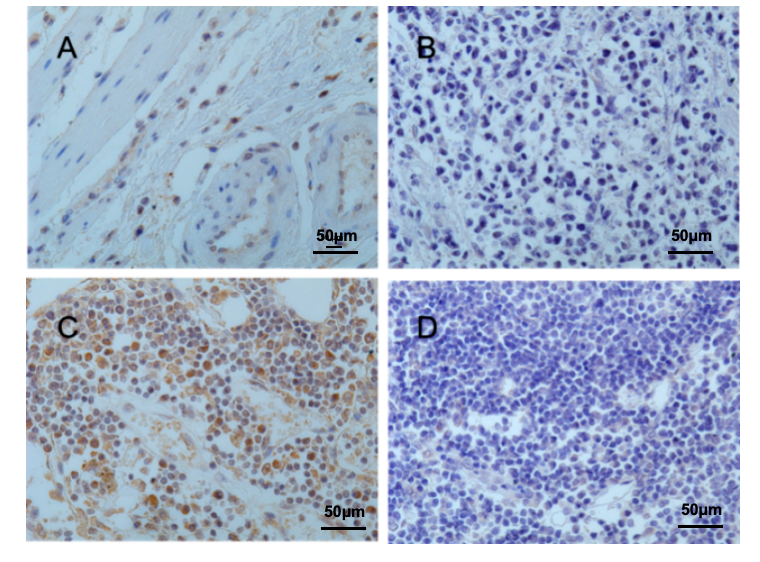


**Figure S1**. Immunohistochemistry results demonstrated the differences between healthy lymph nodes (20 cases) and those of patients with DLBCL (57 cases). (**a**), The high-expression and (**b**) low-expression regions of ZNF382 in the lymph nodes of patients. (**c**) The corresponding high-expression and (**d**) low-expression regions of ZNF382 in healthy individuals. Immunohistochemistry staining was performed according to the protocol of the two-step IHC kit (E-IR-R215, Elabscience) for sample processing and antibody labelling. ZNF382 antibodies were purchased from Abcam (Catalog No. ab25918).

**Table S1**. Protein expression levels of ZNF382 in the DLBCL and control group M (quartile).

| Group | n | OD value | *Z* | P-value |
| --- | --- | --- | --- | --- |
| DLBCL | 57 | 0.0211 (0.0069,0.0609) | -5.056 | <0.001 |
| Control | 20 | 0.1236 (0.0842,0.1889) |  |  |

The results indicated that the ZNF382 expression level in the control group was significantly higher than that in patients with DLBCL (p < 0.001). Analysis of the immunohistochemistry results was performed using Image-Pro Plus 6.0 software, with semi-quantitative measurement of mean optical density (OD) values to represent ZNF382 expression intensity. Statistical analysis of expression levels, which followed a non-normal distribution, was conducted using IBM SPSS 22.0, using the non-Mann–Whitney U test to compare median differences between groups.
